# Supplementary material for: Degradation and detoxification of azo dyes with recombinant ligninolytic enzymes from Aspergillus sp. with secretory overexpression in Pichia pastoris
Source: R Soc Open Sci. 2020 Sep 16;7(9):200688. doi: 10.1098/rsos.200688 (PMC7540776; doi:10.1098/rsos.200688)
Supplement: Supplementary material [file rsos200688supp1.docx]

**Degradation and detoxification of azo dyes with recombinant ligninolytic enzymes from *Aspergillus* sp. with secretory overexpression in *P.pastoris***

Siqi Liu, Xiaolin Xu^*^, Yanshun Kang,

Key Laboratory for Green Processing of Chemical Engineering of Xinjiang Bingtuan/School of Chemistry and Chemical Engineering, Shihezi University, Shihezi 832003, PR China

Corresponding author: Xiaolin Xu

Telephone/Fax: +86-0993-2055015/+86-0993-2057270
E-mail: [xxl_food@shzu.edu.cn](mailto:xxl_food@shzu.edu.cn)

Supporting Information: Figures: 4

**Contents Page**

**Figure S1.** Alignment results of decolorization enzymes genes in NCBI (a: MnP；b: LiP；c: Lac) **2**

**Figure S2.** Verify expression graph (Direct PCR screening of three recombinant *Pichia pastoris* colonies; M: Marker) **2**

**Figure S3.** Enzyme activity of *Aspergillus* sp. TS-A **3**

**Figure S4.** Recombinant enzymes degradation of MY1 and CR (a: Recombinant Lac, LiP, MnP degradation of MY1 and CR; b: Combine recombinant enzymes degradation of CR, Com：LiP + MnP + Lac) **3**

**Figure S1**.Alignment results of decolorization enzymes genes in NCBI (a: Lac；b:MnP；c: LiP)


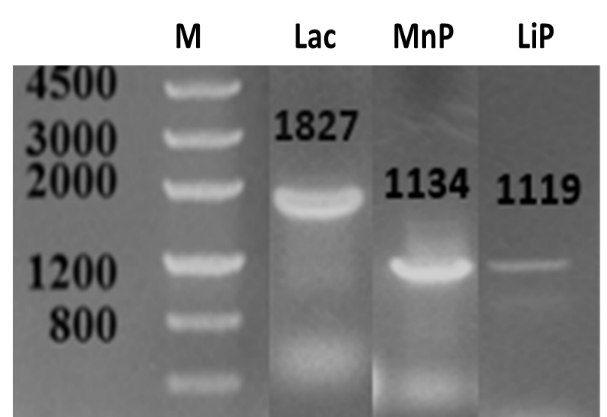


**Figure S2**. Verify expression graph (Direct PCR screening of three recombinant *Pichia pastoris* colonies; M: Marker)

**Figure S3**. Enzyme activity of *Aspergillus* sp. TS-A

**Figure S4**. Recombinant enzymes degradation of MY1 and CR (a: Recombinant Lac, LiP, MnP degradation of MY1 and CR; b: Combine recombinant enzymes degradation of CR, Com：LiP + MnP + Lac)
