# Supplementary material for: Degradation and detoxification of azo dyes with recombinant ligninolytic enzymes from Aspergillus sp. with secretory overexpression in Pichia pastoris
Source: R Soc Open Sci. 2020 Sep 16;7(9):200688. doi: 10.1098/rsos.200688 (PMC7540776; doi:10.1098/rsos.200688)
Supplement: Supporting date [file rsos200688supp2.docx]

1. OD600

Lac

| Time | OD600 |  |  |  |  |
| --- | --- | --- | --- | --- | --- |
| h | 1 | 2 | 3 | average | STDEV.P |
| 0 | 0 | 0 | 0 | 0 | 0 |
| 4 | 0.03 | 0.23 | 0.071 | 0.110333 | 0.086257 |
| 8 | 0.062 | 0.18 | 0.339 | 0.193667 | 0.113497 |
| 12 | 0.079 | 0.23 | 1.269 | 0.526 | 0.528985 |
| 16 | 1.137 | 1.891 | 1.576 | 1.534667 | 0.309204 |
| 20 | 2.174 | 2.479 | 2.947 | 2.533333 | 0.317906 |
| 24 | 2.281 | 2.642 | 3.977 | 2.966667 | 0.729457 |
| 28 | 6.198 | 5.427 | 6.645 | 6.09 | 0.503077 |
| 32 | 7.024 | 7.419 | 7.92 | 7.454333 | 0.366643 |
| 36 | 9.301 | 8.511 | 9.819 | 9.210333 | 0.537824 |
| 40 | 10.287 | 9.95 | 10.993 | 10.41 | 0.434595 |
| 44 | 11.302 | 10.9 | 12.409 | 11.537 | 0.638064 |
| 48 | 11.414 | 11.21 | 12.495 | 11.70633 | 0.563856 |

Mnp

| 0 | 0 | 0 | 0 | 0 | 0 |
| --- | --- | --- | --- | --- | --- |
| 4 | 0.09 | 0.111 | 0.109 | 0.103333 | 0.009463 |
| 8 | 0.15 | 0.157 | 0.148 | 0.151667 | 0.003859 |
| 12 | 0.298 | 0.32 | 0.311 | 0.309667 | 0.009031 |
| 16 | 0.995 | 1.089 | 1.051 | 1.045 | 0.038609 |
| 20 | 1.719 | 1.72 | 1.944 | 1.794333 | 0.105831 |
| 24 | 2.51 | 2.469 | 2.551 | 2.51 | 0.033476 |
| 28 | 3.898 | 4.019 | 4.407 | 4.108 | 0.217119 |
| 32 | 6.593 | 6.477 | 6.91 | 6.66 | 0.18301 |
| 36 | 8.671 | 8.512 | 8.179 | 8.454 | 0.205002 |
| 40 | 8.978 | 9.157 | 8.881 | 9.005333 | 0.114322 |
| 44 | 9.679 | 10.39 | 9.819 | 9.962667 | 0.307528 |
| 48 | 9.892 | 10.329 | 10.321 | 10.18067 | 0.204144 |

Lip

| 0 | 0 | 0 | 0 | 0 | 0 |
| --- | --- | --- | --- | --- | --- |
| 4 | 0.099 | 0.101 | 0.101 | 0.100333 | 0.000943 |
| 8 | 0.157 | 0.15 | 0.148 | 0.151667 | 0.003859 |
| 12 | 0.301 | 0.319 | 0.296 | 0.305333 | 0.009877 |
| 16 | 1.087 | 1.139 | 1.131 | 1.119 | 0.022862 |
| 20 | 1.674 | 1.809 | 2.21 | 1.897667 | 0.227626 |
| 24 | 2.693 | 2.899 | 2.489 | 2.693667 | 0.167382 |
| 28 | 4.535 | 4.771 | 4.196 | 4.500667 | 0.235995 |
| 32 | 6.867 | 7.099 | 6.41 | 6.792 | 0.286239 |
| 36 | 8.819 | 8.611 | 8.994 | 8.808 | 0.156552 |
| 40 | 9.209 | 9.397 | 8.735 | 9.113667 | 0.278541 |
| 44 | 9.89 | 10.327 | 9.857 | 10.02467 | 0.214206 |
| 48 | 9.901 | 10.221 | 9.804 | 9.975333 | 0.178169 |

2. Protein concentration

Lac

| time | A1 | C1 | A2 | C2 | A3 | C3 | A_a_ | C_a_ | STDEV.P |
| --- | --- | --- | --- | --- | --- | --- | --- | --- | --- |
| 0 | 0 | 0 | 0 | 0 | 0 | 0 | 0 | 0 | 0 |
| 24 | 0.029 | 0.010 | 0.073 | 0.025 | 0.117 | 0.040 | 0.073 | 0.025 | 0.012 |
| 48 | 0.088 | 0.030 | 0.147 | 0.050 | 0.205 | 0.070 | 0.147 | 0.050 | 0.016 |
| 72 | 0.226 | 0.077 | 0.147 | 0.050 | 0.285 | 0.097 | 0.219 | 0.075 | 0.019 |
| 96 | 0.246 | 0.084 | 0.241 | 0.082 | 0.344 | 0.117 | 0.277 | 0.094 | 0.016 |
| 120 | 0.235 | 0.080 | 0.346 | 0.118 | 0.317 | 0.108 | 0.299 | 0.102 | 0.016 |

Lip

| 0 | 0 | 0 | 0 | 0 | 0 | 0 | 0 | 0 | 0 |
| --- | --- | --- | --- | --- | --- | --- | --- | --- | --- |
| 24 | 0.180 | 0.061 | 0.179 | 0.061 | 0.193 | 0.066 | 0.184 | 0.063 | 0.002 |
| 48 | 0.311 | 0.106 | 0.370 | 0.126 | 0.305 | 0.104 | 0.329 | 0.112 | 0.010 |
| 72 | 0.463 | 0.158 | 0.516 | 0.176 | 0.451 | 0.154 | 0.477 | 0.163 | 0.010 |
| 96 | 0.543 | 0.185 | 0.522 | 0.178 | 0.587 | 0.200 | 0.551 | 0.188 | 0.009 |
| 120 | 0.574 | 0.196 | 0.692 | 0.236 | 0.605 | 0.206 | 0.624 | 0.213 | 0.017 |

Mnp

| 0 | 0 | 0 | 0 | 0 | 0 | 0 | 0 | 0 | 0 |
| --- | --- | --- | --- | --- | --- | --- | --- | --- | --- |
| 24 | 0.255 | 0.087 | 0.150 | 0.051 | 0.258 | 0.088 | 0.220 | 0.075 | 0.017 |
| 48 | 0.408 | 0.139 | 0.464 | 0.158 | 0.493 | 0.168 | 0.440 | 0.155 | 0.012 |
| 72 | 0.525 | 0.179 | 0.508 | 0.173 | 0.502 | 0.171 | 0.514 | 0.174 | 0.003 |
| 96 | 0.643 | 0.219 | 0.698 | 0.238 | 0.725 | 0.247 | 0.690 | 0.235 | 0.012 |
| 120 | 0.971 | 0.331 | 0.901 | 0.307 | 0.877 | 0.299 | 0.917 | 0.312 | 0.014 |

3. enzyme activity

| time | k=A/T | EA | STDEV.P | k=A/T | EA | STDEV.P | k=A/T | EA | STDEV.P |
| --- | --- | --- | --- | --- | --- | --- | --- | --- | --- |
| 0 | 0 | 0 | 0 | 0 | 0 | 0 | 0 | 0 | 0 |
| 24 | 0.002212 | 14.75 | 2.9 | 0.068858 | 42.375 | 3.1 | 0.107558 | 46.25 | 2.8 |
| 48 | 0.003562 | 23.75 | 3.4 | 0.127966 | 78.75 | 2.5 | 0.163517 | 70.3125 | 3.4 |
| 72 | 0.003994 | 26.625 | 3 | 0.142184 | 87.5 | 2.1 | 0.181395 | 78 | 2.9 |
| 96 | 0.005238 | 34.92 | 2.9 | 0.219573 | 135.125 | 3 | 0.239826 | 103.125 | 2.3 |

4. temperture

| Temperture | k=A/T | EA | STDEV.P | k=A/T | EA | STDEV.P | k=A/T | EA | STDEV.P |
| --- | --- | --- | --- | --- | --- | --- | --- | --- | --- |
| 25 | 0.154371 | 95 | 2 | 0.183721 | 79 | 3 | 0.00375 | 25 | 1 |
| 30 | 0.227494 | 140 | 4 | 0.22093 | 95 | 5 | 0.0048 | 32 | 3 |
| 35 | 0.165746 | 102 | 2 | 0.244186 | 105 | 6.5 | 0.00525 | 35 | 3.5 |
| 40 | 0.129997 | 80 | 3 | 0.186047 | 80 | 1 | 0.00375 | 25 | 1 |
| 45 | 0.123497 | 76 | 1 | 0.174419 | 75 | 3 | 0.003 | 20 | 2 |
| 50 | 0.121872 | 75 | 1 | 0.116279 | 50 | 2 | 0.0027 | 18 | 2 |
| 60 | 0.097498 | 60 | 2 | 0.093023 | 40 | 2 | 0.0021 | 14 | 3 |

5. pH

| pH | k=A/T | EA | STDEV.P | k=A/T | EA | STDEV.P | k=A/T | EA | STDEV.P |
| --- | --- | --- | --- | --- | --- | --- | --- | --- | --- |
| 1 | 0.048749 | 30 | 2 | 0.046512 | 20 | 4 | 0.0012 | 8 | 2 |
| 3 | 0.214495 | 132 | 3 | 0.206977 | 89 | 2 | 0.00435 | 29 | 3 |
| 5 | 0.194995 | 120 | 4 | 0.27907 | 120 | 6 | 0.00345 | 23 | 1 |
| 7 | 0.116997 | 72 | 2 | 0.134884 | 58 | 1 | 0.003 | 20 | 2 |
| 9 | 0.058499 | 36 | 2 | 0.086047 | 37 | 2 | 0.0015 | 10 | 1 |

6. metal ions

Lac

| metal ions | Fe |  |  | Mg |  |  | Ca |  |  | Cu |  |  | Zn |  |  |
| --- | --- | --- | --- | --- | --- | --- | --- | --- | --- | --- | --- | --- | --- | --- | --- |
|  | k | EA | ST | k | EA | ST | k | EA | ST | K | EA | ST | k | EA | ST |
| 0 | 0.00375 | 25 | 2.5 | 0.00405 | 27 | 3 | 0.00495 | 33 | 2.5 | 0.003 | 20 | 4 | 0.00375 | 25 | 3 |
| 0.5 | 0.00465 | 31 | 5 | 0.00465 | 31 | 4.5 | 0.006 | 40 | 4.5 | 0.00495 | 33 | 3 | 0.00465 | 31 | 4.5 |
| 1 | 0.0066 | 44 | 4.5 | 0.00375 | 25 | 2 | 0.00465 | 31 | 2.5 | 0.0084 | 56 | 3 | 0.004875 | 32.5 | 2.5 |
| 1.5 | 0.0042 | 28 | 3 | 0.003225 | 21.5 | 2.5 | 0.002625 | 17.5 | 3 | 0.004875 | 32.5 | 3 | 0.003225 | 21.5 | 3 |
| 2 | 0.003225 | 21.5 | 2.7 | 0.002625 | 17.5 | 3 | 0.00225 | 15 | 2.5 | 0.00345 | 23 | 2 | 0.002625 | 17.5 | 2.5 |
| 2.5 | 0.00375 | 25 | 2.5 | 0.0015 | 10 | 2.5 | 0.001725 | 11.5 | 1.5 | 0.00375 | 25 | 2.5 | 0.002625 | 17.5 | 2 |

Lip

| 0 | 0.232558 | 100 | 6 | 0.24186 | 104 | 4 | 0.274419 | 118 | 3 | 0.274419 | 118 | 1 | 0.223256 | 96 | 3 |
| --- | --- | --- | --- | --- | --- | --- | --- | --- | --- | --- | --- | --- | --- | --- | --- |
| 0.5 | 0.232558 | 100 | 5 | 0.460465 | 198 | 4 | 0.362791 | 156 | 3 | 0.25814 | 111 | 2 | 0.209302 | 90 | 2 |
| 1 | 0.24186 | 104 | 2 | 0.283721 | 122 | 3 | 0.362791 | 156 | 1 | 0.232558 | 100 | 2 | 0.17907 | 77 | 3 |
| 1.5 | 0.209302 | 90 | 1 | 0.255814 | 110 | 3 | 0.232558 | 100 | 2 | 0.186047 | 80 | 2 | 0.169767 | 73 | 1 |
| 2 | 0.153488 | 66 | 2 | 0.232558 | 100 | 1 | 0.186047 | 80 | 6 | 0.169767 | 73 | 1 | 0.153488 | 66 | 2 |
| 2.5 | 0.123953 | 53.3 | 1 | 0.169767 | 73 | 2 | 0.186047 | 80 | 1.5 | 0.081395 | 35 | 4 | 0.093023 | 40 | 1 |

Mnp

| 0 | 0.158434 | 97.5 | 5 | 0.182808 | 112.5 | 2 | 0.158434 | 97.5 | 3 | 0.170621 | 105 | 5 | 0.121872 | 75 | 5 |
| --- | --- | --- | --- | --- | --- | --- | --- | --- | --- | --- | --- | --- | --- | --- | --- |
| 0.5 | 0.20312 | 125 | 5 | 0.251869 | 155 | 3 | 0.243744 | 150 | 2 | 0.243744 | 150 | 3 | 0.251869 | 155 | 5 |
| 1 | 0.20312 | 125 | 5 | 0.194995 | 120 | 2 | 0.292493 | 180 | 1 | 0.243744 | 150 | 5 | 0.268118 | 165 | 3 |
| 1.5 | 0.182808 | 112.5 | 3 | 0.170621 | 105 | 2 | 0.243744 | 150 | 2 | 0.146246 | 90 | 2 | 0.182808 | 112.5 | 2.5 |
| 2 | 0.109685 | 67.5 | 2 | 0.121872 | 75 | 5 | 0.20312 | 125 | 3 | 0.136497 | 84 | 3 | 0.134059 | 82.5 | 2.5 |
| 2.5 | 0.121872 | 75 | 2 | 0.073123 | 45 | 2 | 0.158434 | 97.5 | 1 | 0.079217 | 48.75 | 2 | 0.121872 | 75 | 3 |

7. degradation of dye

| degradation | Lac |  |  | A | S | Lip |  |  | A | S | Mnp |  |  | A | S |
| --- | --- | --- | --- | --- | --- | --- | --- | --- | --- | --- | --- | --- | --- | --- | --- |
| Congo Red | 45.5 | 45.57 | 45.41 | 45.49333 | 0.06549 | 37.377 | 37.5 | 37.557 | 37.478 | 0.075113 | 39.24 | 39.15 | 39.37 | 39.25333 | 0.090308 |
| Mordant Yellow 1 | 4.54 | 6.57 | 5.7 | 5.603333 | 0.831558 | 3.3 | 3.05 | 2.95 | 3.1 | 0.147196 | 3.4 | 3.77 | 3.9 | 3.69 | 0.211818 |
| Disperse Blue 2BLN | 9.73 | 9.87 | 9.15 | 9.583333 | 0.311698 | 5 | 4.839 | 4.56 | 4.799667 | 0.18177 | 3.26 | 3.9 | 3.15 | 3.436667 | 0.33069 |
| Bromophenol Blue | 72.164 | 73.79 | 74.903 | 73.619 | 1.124711 | 68.7 | 67.1 | 66.9 | 67.56667 | 0.805536 | 65 | 69.65 | 63.2 | 65.95 | 2.717536 |

8. Toxicity Assay

| water |  |  |  | A | S |
| --- | --- | --- | --- | --- | --- |
| shoot | 7.5 | 8 | 8.4 | 7.966667 | 0.368179 |
| root | 6.5 | 5.8 | 7.2 | 6.5 | 0.571548 |
| leaf | 2.3 | 2.4 | 2.5 | 2.4 | 0.08165 |
| weight | 0.1948 | 0.2133 | 0.1832 | 0.1971 | 0.012395 |

| CR |  |  |  |  |  |
| --- | --- | --- | --- | --- | --- |
| shoot | 5 | 5 | 5.3 | 5.1 | 0.141421 |
| root | 3.5 | 3.5 | 4 | 3.666667 | 0.235702 |
| leaf | 1.3 | 1.2 | 1.5 | 1.333333 | 0.124722 |
| weight | 0.1353 | 0.128 | 0.155 | 0.139433 | 0.011404 |

| Lac |  |  |  |  |  |
| --- | --- | --- | --- | --- | --- |
| shoot | 6.2 | 6 | 7 | 6.4 | 0.432049 |
| root | 5 | 6 | 4 | 5 | 0.816497 |
| leaf | 2 | 2.1 | 1.8 | 1.966667 | 0.124722 |
| weight | 0.1633 | 0.186 | 0.1561 | 0.168467 | 0.012742 |

| Lip |  |  |  |  |  |
| --- | --- | --- | --- | --- | --- |
| shoot | 10 | 6.6 | 8.5 | 8.366667 | 1.391242 |
| root | 7.2 | 6.5 | 6 | 6.566667 | 0.492161 |
| leaf | 2.5 | 2.3 | 2.2 | 2.333333 | 0.124722 |
| weight | 0.17 | 0.2738 | 0.1947 | 0.212833 | 0.044274 |

| Mnp |  |  |  |  |  |
| --- | --- | --- | --- | --- | --- |
| shoot | 5 | 5 | 5.4 | 5.133333 | 0.188562 |
| root | 5.5 | 5.7 | 5.5 | 5.566667 | 0.094281 |
| leaf | 1.9 | 1.6 | 2 | 1.833333 | 0.169967 |
| weight | 0.2146 | 0.1819 | 0.1808 | 0.192433 | 0.015681 |

| Com |  |  |  |  |  |
| --- | --- | --- | --- | --- | --- |
| shoot | 10 | 7 | 7 | 8 | 1.414214 |
| root | 6.5 | 7.2 | 5.7 | 6.466667 | 0.612826 |
| leaf | 2.5 | 2.3 | 2.3 | 2.366667 | 0.094281 |
| weight | 0.186 | 0.1947 | 0.2146 | 0.198433 | 0.011971 |

9. sequence

1. Lac

ATGGCTACTGTTTCTTTGCCAGCTAACATCAACACTGCTATGGCTTTGGACATCGGTATCATCTCTCAACCATCTCCATTGCAAAGATTGATCCCATCTAGAGTTTCTACTAACCAAATCCACCCAACTGTTCACGAACCAGACGTTTCTAGACCATTGATCGAATTGCACCCAGAAGACCACATCTACAGAAACCCATCTACTCAACACCACGACTGGGTTGTTACTGCTGACCACAGAAGACCAGACGGTGTTTTGAAGAGAGTTTACTTGATCAACGGTTTGTTCCCAGGTCCAACTGTTGAAGCTAGATCTGGTGACAGATTGATCGTTAACGTTACTAACTCTTTGGAAGAAGAACCAATCTCTATCCACTGGCACGGTATCCACATCGAATCTAAGTCTTCTGCTATGTTGTGGATGGAACCATTGGTTATGGACGGTGCTGTTGGTGTTACTCAAAGAGCTATCCCACCAGGTTCTACTTTCACTTACAACTTCACTATCCCAACTGACCAATCTGGTACTTTCTGGTACCACGCTCACTCTGGTTTGTTGAGAGCTGACGGTTTGTACGGTGGTTTGATCGTTCACGAACCATCTCCAAAGTCTACTGTTAGAGGTTTGTTGGCTAGAGCTGACCAACAAGAATTGGGTTCTTACGACAAGGACATCTTGTTGTTGGTTGGTGACTGGTACCACAGATCTGCTGACCAAGTTTACTCTTGGTACATGAGAGCTGGTTCTTTCGGTAACGAACCAGTTCCAGACTCTTTGTTGATCAACGGTGTTGGTCACTTCGACTGTTCTATGGCTGTTCCAGCTAGACCAGTTGACTGTATCTTGAGACACATGAACGTTTCTTACTTGGACGCTAAGGGTGACGCTGCTTACAGAGTTAGAGTTGTTAACACTGGTTCTGTTGCTGGTTTCACTTTGGGTTTCCAAAACAGAGAATTTTCTTTGATCCAAGTTGACAACATCGACGTTGAACAACAAGACTCTAACTCTGCTGGTGTTTTGTACCCAGGTCAAAGAATGGACATCATCTTGAGACCATCTCCAGAAAAGGCTCCATCTTCTTTGACTATCGACTTGGACAAGGAATGTTTCAGATACCCAAACCCAGCTTTGACTTCTGTTCAAACTTACAACATCAAGAAGTCTCCAAACAACTTGGCTCCAACTATCTCTCCATCTAACAACACTATCTCTTTGTCTGAAGTTGCTACTAGAAAGTCTTTGTTGTCTGGTTTGCCAGCTAACTCTCACCAAACTTACGTTGTTTACACTAAGATCGAAAAGTTGTCTATCAACCACAACGTTCCATACGGTTTCTTCAACAGAACTTCTTGGAGACCACAAATCGACACTCCATTGATCGACTTGCCAAGAGAAGAATGGGACGAAAACCAATTGGTTTTGTCTACTGGTTCTACTACTTCTAGACCATTGTCTTCTGAACACGACCAAGACTTGTGGATCGACTTGGTTGTTAACAACTTGGACGACTCTGGTCACCCATTCCACTTCACTCACGGTCACCACTTCTACATCTTGAGAACTTACCAAGCTCCAGTTGGTTGGGGTGCTTACAACCCATTCACTGACGCTCACCCACCAGGTTTGGCTTTGTCTTCTGGTTCTTCTTCTAAGGCTGACTCTCCATACGACTTGTCTAGAGCTCAATTGAGAGACACTGTTTACATCCCATCTAGAGGTCACGCTGTTTTGAGATTCAGAGCTGACAACCCAGGTATCTGGTTGTTCCACTGTCACATCATCTGGCACCAAGCTTCTGGTATGGCTATGTTGTTGCAAATGTAA

MATVSLPANINTAMALDIGIISQPSPLQRLIPSRVSTNQIHPTVHEPDVSRPLIELHPEDHIYRNPSTQHHDWVVTADHRRPDGVLKRVYLINGLFPGPTVEARSGDRLIVNVTNSLEEEPISIHWHGIHIESKSSAMLWMEPLVMDGAVGVTQRAIPPGSTFTYNFTIPTDQSGTFWYHAHSGLLRADGLYGGLIVHEPSPKSTVRGLLARADQQELGSYDKDILLLVGDWYHRSADQVYSWYMRAGSFGNEPVPDSLLINGVGHFDCSMAVPARPVDCILRHMNVSYLDAKGDAAYRVRVVNTGSVAGFTLGFQNREFSLIQVDNIDVEQQDSNSAGVLYPGQRMDIILRPSPEKAPSSLTIDLDKECFRYPNPALTSVQTYNIKKSPNNLAPTISPSNNTISLSEVATRKSLLSGLPANSHQTYVVYTKIEKLSINHNVPYGFFNRTSWRPQIDTPLIDLPREEWDENQLVLSTGSTTSRPLSSEHDQDLWIDLVVNNLDDSGHPFHFTHGHHFYILRTYQAPVGWGAYNPFTDAHPPGLALSSGSSSKADSPYDLSRAQLRDTVYIPSRGHAVLRFRADNPGIWLFHCHIIWHQASGMAMLLQM*

2. Mnp

ATGGCTTTCGCTTCTTTGTTCACTTTGGTTGTTTTGGCTGCTGTTTCTAACGCTGCTCCAACTGCTGTTTGTGCTGACGGTACTAGAGTTTCTAACGCTGCTTGTTGTGCTTTCATCCCATTGGCTCAAGACTTGCACGAAACTTTGTTCATGGGTGACTGTGGTGAAGACGCTCACGAAGTTATCAGATTGACTTTCCACGACGCTGTTGCTATCTCTTCTTCTATGGGTCCATCTGCTGGTGGTGGTGCTGACGGTTCTATGTTGTTGTTCCCAACTGTTGAACCAAACTTCTCTGCTAACAACGGTATCGACGACTCTGTTAACAACTTGATCCCATTCTTGTCTAAGCACGCTGTTTCTGCTGGTGACTTGGTTCAATTCGCTGGTGCTGTTGCTTTGACTAACTGTCCAGGTGCTCCACAATTGGAATTTTTGGCTGGTAGACCAAACCACACTATCGCTGCTATCGACGGTTTGATCCCAGAACCACAAGACGACGTTACTAAGATCTTGGCTAGATTCGAAGACGCTGGTGGTTTCTCTCCATTCGAAGTTGTTTCTTTGTTGGCTTCTCACACTGTTGCTAGAGCTGACAAGGTTGACGGTACTATCGACGCTGCTCCATTCGACTCTACTCCATTCACTTTCGACACTCAAGTTTTTTTGGAAGTGTTATTGAAGGGCACTGGCTTCCCAGGTACTAATAACAACACTGGTGAAGTTGCTTCTCCATTGCCATTGACTTCTGGTAACGACACTGGTGAAATGAGATTGCAATCTGACTTCGCTTTGGCTAGAGACGAAAGAACTGCTTGTTTCTGGCAATCTTTCGTTAACGAACAAGAATTTATGGCTCAATCTTTCAAGGCTGCTATGTCTAAGTTGGCTGTTTTGGGTCACTCTAGATCTTCTTTGGTTGACTGTTCTGACGTTGTTCCAGCTCCAAAGCCAGCTGTTAACAAGCCAGCTACTTTCCCAGCTACTACTGGTCCAGACGACTTGGAATTGACTTGTACTGCTGAAAGATTCCCAACTTTGTCTGTTGACCCAGGTGCTCAGCAAACTCTCATCCCACACTGCTCTGACGGTGACCAAGTTTGTGCTACTGTTCAATTCACTGGTCCAGCTTAA

MAFASLFTLVVLAAVSNAAPTAVCADGTRVSNAACCAFIPLAQDLHETLFMGDCGEDAHEVIRLTFHDAVAISSSMGPSAGGGADGSMLLFPTVEPNFSANNGIDDSVNNLIPFLSKHAVSAGDLVQFAGAVALTNCPGAPQLEFLAGRPNHTIAAIDGLIPEPQDDVTKILARFEDAGGFSPFEVVSLLASHTVARADKVDGTIDAAPFDSTPFTFDTQVFLEVLLKGTGFPGTNNNTGEVASPLPLTSGNDTGEMRLQSDFALARDERTACFWQSFVNEQEFMAQSFKAAMSKLAVLGHSRSSLVDCSDVVPAPKPAVNKPATFPATTGPDDLELTCTAERFPTLSVDPGAQQTLIPHCSDGDQVCATVQFTGPA*

3. Lip

ATGGCTTTCAAGAGATTGTTGGCTGTTTTGACTGCTGCTATCTCTTTGGGTGCTGTTCAAGGTGTTGCTGTTGAAAAGAGAGCTACTTGTTCTAACGGCAAGACTGTAAGCGCTTCTTCTTGCTGTGCTTGGTTCAACGTGCTCTCTGACATCCAAGAAAACTTGTTCAACGGTGGTCAATGTGGTGCTGAAGCTCACGAATCTATACGTTTGGTGTTCCACGACAGCATCGCTATCTCTCCAGCTATGGAAGCTGCTGGTCAATTCGGTGGTGGTGGTGCTGACGGTTCTATCATGATCTTCGACGAAATCGAAACTAACTTCCACCCAAACATCGGTTTGGACGAAATCGTTAGATTGCAAAAGCCATTCGTTCAAAAGCACGGTGTTACTCCAGGTGACTTCATCGCTTTCGCTGGTGCTGTTGCTTTGTCTAACTGTCCAGGTGCTCCACAAATGAACTTCTTCACTGGTAGAGCTCCAGCTACTCAAGCTGCTCCAGACGGTTTGGTTCCAGAACCATTCCACACTGTTGACCAAATCATCGACAGAGTTGGTGACGCTGGTGAATTTGACGAATTGGAATTGGTTTGGATGTTGTCTGCTCACTCTATCGCTGCTGCTAACGACGTTGACCCAACTACTCAAGGTTTGCCATTCGACTCTACTCCAGGTATCTTCGACTCTCAATTCTTCGTTGAAACTCAATTGGCTGGTACTGGTTTCCCAGCTTCTGCTAACAACCAAGGTGAAGTTACTTCTCCATTGGCTGGTGAAATGAGATTGCAATCTGACTTCTTGATCGCTAGAGACGCTAGAACTGCTTGTGAATGGCAATCTTTCGTTAACAACCAAAGTAAGTTGGTAAGCGACTTCCAATTCATCTTCTTGGCTTTGACTCAATTGGGTCAAGACCCAACTGTTATGACTGACTGTTCTGACGTTATCCCAATCTCTAAGCCAGCTCCAGCTAACACTCCAGGTTTCTCTTTCTTCCCAGCTGGTAAGACTATGGCTGACGTTGAACAAGCTTGTGCTGAAACTCCATTCCCAACTTTGTCTACTTTGCCAGGTCCACAAACTTCTGTTGCTAGAATCCAACCACCACCAGGTGCTTAA

MAFKRLLAVLTAAISLGAVQGVAVEKRATCSNGKTVSASSCCAWFNVLSDIQENLFNGGQCGAEAHESIRLVFHDSIAISPAMEAAGQFGGGGADGSIMIFDEIETNFHPNIGLDEIVRLQKPFVQKHGVTPGDFIAFAGAVALSNCPGAPQMNFFTGRAPATQAAPDGLVPEPFHTVDQIIDRVGDAGEFDELELVWMLSAHSIAAANDVDPTTQGLPFDSTPGIFDSQFFVETQLAGTGFPASANNQGEVTSPLAGEMRLQSDFLIARDARTACEWQSFVNNQSKLVSDFQFIFLALTQLGQDPTVMTDCSDVIPISKPAPANTPGFSFFPAGKTMADVEQACAETPFPTLSTLPGPQTSVARIQPPPGA*
